# Digital Storytelling: The New Arts-Based Research Method

**DOI:** 10.18502/ijph.v49i7.3602

**Published:** 2020-07

**Authors:** Sahar KHOSHKESHT, Alireza NIKBAKHT NASRABADI, Leila MARDANIAN DEHKORDI

**Affiliations:** School of Nursing & Midwifery, Tehran University of Medical Sciences, Tehran, Iran

## Dear Editor-in-Chief

In the modern world, the use of digital technology is growing rapidly; people documented their lives more and more on social networks. Therefore, in keeping with this wave of technology, researchers should use new research methods that can interact with users such as dance, poetry, theater, body mapping and digital storytelling (1). Digital storytelling (DST) is an innovative, collaborative, arts-based research method that including storytelling, teamwork, and technology to facilitate the creation of 2 to 3 min video clips with a combination of photo, sound and other audio devices featuring personal stories (1,2).

Nevertheless, DST can be used in the various areas such 1- Education, training or professional development 2- Knowledge translation 3- Treatment 4- Community development 5- Preserving cultural heritage, and 6- Research, especially in the field of qualitative studies, to hear participants' voices (3). Health researchers are increasingly using DST in their research. In this letter; we draw the attention of researchers to the basic understanding and application of DST in field of medical research.

DST can be used as an independent research method or complementary to other methods. It can also be used as a tool in interviews or focus groups (4). As a new data collection technique, it can provide opportunity for participants to be active and reflective. The nature of communication in the DST process reflects the participatory-centered approach (2). This innovative research method helps to understand the subtle meaning of participants' experiences in research, to explain complex stories, and to share findings in an attractive way (5). Ultimately, the sharing of experiences and knowledge transfer in this way can improve the quality of care (2).

Although DST is ideal in participatory research, it can be conducted in a non-participatory manner (6). In addition, DST also can be an appropriate research method for other marginal groups including refugees, immigrants (7) and people with special circumstances such as disabilities or low literacy (8). The benefits of DST are beyond the narrative of the story which include participation in research, knowledge translation, interdisciplinary research, evaluating the time and efforts in research (3).

The nature of audio-visual of DST is used to examine sensitive phenomena and displays sensory information that is not accessible via text with an interview, and participants can express their stories in multidimensional way. On the other hand, due to the active participation of the participant in the research process, it creates a nearly flat relationship between the researcher, the participant and the stakeholders (9).

There are some challenges to using DST. To portray some of the difficult events may be painful. In addition, DST requires time, resources and as training. The use of the real name of the participants and the privacy of individuals, attention to the ethical aspects of this method and the quality of the production of artwork is also challenging (10).

Finally, we know that storytelling is a universal and powerful way of making meaning and knowledge transfer. Stories are used in the therapeutic, educational, and research areas. Therefore, we need to know and respect it more and before.
